# Atomic-scale mapping of dipole frustration at 90° charged domain walls in ferroelectric PbTiO_3_ films

**DOI:** 10.1038/srep04115

**Published:** 2014-02-18

**Authors:** Y. L. Tang, Y. L. Zhu, Y. J. Wang, W. Y. Wang, Y. B. Xu, W. J. Ren, Z. D. Zhang, X. L. Ma

**Affiliations:** 1 Shenyang National Laboratory for Materials Science, Institute of Metal Research, Chinese Academy of Sciences, Wenhua Road 72, 110016 Shenyang, China; 2 These authors contributed equally to this work.

## Abstract

The atomic-scale structural and electric parameters of the 90° domain-walls in tetragonal ferroelectrics are of technological importance for exploring the ferroelectric switching behaviors and various domain-wall-related novel functions. We have grown epitaxial PbTiO_3_/SrTiO_3_ multilayer films in which the electric dipoles at 90° domain-walls of ferroelectric PbTiO_3_ are characterized by means of aberration-corrected scanning transmission electron microscopy. Besides the well-accepted head-to-tail 90° uncharged domain-walls, we have identified not only head-to-head positively charged but also tail-to-tail negatively charged domain-walls. The widths, polarization distributions, and strains across these charged domain-walls are mapped quantitatively at atomic scale, where remarkable difference between these domain-walls is presented. This study is expected to provide fundamental information for understanding numerous novel domain-wall phenomena in ferroelectrics.

Ferroelectrics possess controllable polar states and electromechanical couplings, they were found extensive applications as high-density memories, thin-film capacitors and actuators as well as sensors^1^,^2^,^3^. In addition, they are showing multifunctional capabilities, as seen the finding of domain-wall conductivity^3^,^4^,^5^,^6^ in ferroelectrics. Domain-walls in ferroelectrics are topological interfaces that separate domains with different orientations of polarizations. Historically, the domain-walls in ferroelectrics were thought to be simple, but their physical nature is found to be quite complicated in the past decade^3^. The local structural, chemical, and electric features as well as the dipole-defect interactions of domain-walls are of great importance and the macroscopic physical properties of ferroelectrics are strongly associated with these microstructural characteristics^7^,^8^,^9^,^10^,^11^,^12^.

Tetragonal ferroelectrics generally exhibit two types of domain-walls: 90° and 180° domain-walls, which have dipoles across the domain-walls arranged as 90° (nearly) and 180° configurations, respectively^7^,^8^,^9^,^10^,^11^,^12^,^13^,^14^. The 180° domains have the same strain, and hence are easy to switch^8^,^10^,^11^,^12^. However, the strong coupling of the polarization to the elastic strain of 90° domains limits the poling and piezoelectric ability of tetragonal ferroelectrics, which is a large obstacle for their potentials^7^,^8^,^9^,^10^,^11^,^12^. To understand the atomistic mechanisms involved during 90° domains switching, it is highly essential to figure out the structural and electric behaviors of 90° domain-walls on the atomic-scale which is known little up to date. Previously, the width of 90° domain-walls was studied by conventional transmission electron microscopy (TEM), and was 20 nm for BaTiO_3_ (ref. 15), then 4–15 nm for BaTiO_3_, (Ba, Pb)TiO_3_ and Pb(Zr_0.52_Ti_0.48_)O_3_ (refs. 16,17,18). These measurements might be overestimated due to the limitation of instrument resolution, since the width of 90° domain-walls in PbTiO_3_ were later found to be atomically sharp as 1.0–2.8 nm, determined by high resolution transmission electron microscopy (HRTEM) and weak beam transmission electron microscopy^19^,^20^,^21^,^22^. In the meanwhile, more and more theoretical works have proposed the presence of atomically sharp 90° domain-walls in tetragonal ferroelectrics^22^,^23^,^24^. Nevertheless, the dipoles across the 90° domain-walls were almost ignored in previous experiments, although the appearance of ‘head-to-head' domain-walls was inferred in some modified rhombohedral PZT ceramics by the diffraction contrast analysis in a TEM^25^. Generally, the dipole configurations across the 90° domain-walls were arbitrarily treated as ‘head-to-tail' arrangement in theoretical simulations based on the consideration of the electrostatic energy^15^,^16^,^17^,^18^,^19^,^20^,^21^,^22^,^23^,^24^. The newly developed scanning probe microscopy (SPM) based instruments show great potential to map the polarization distribution at ferroelectric surfaces, but its lateral resolution is about 5–30 nm, which is far from atomic-scale^3^,^26^,^27^.

The atomic and electronic behaviors of ferroelectrics have become readily accessible through aberration-corrected scanning transmission electron microscopy (STEM)^28^,^29^ and accurate mapping of order-parameter fields such as lattice^30^,^31^, polarization^14^,^32^,^33^,^34^ and octahedral tilts^35^,^36^,^37^ has recently become feasible. In this study, we have grown PbTiO_3_/SrTiO_3_ multilayer films and mapped atomic details and polarization distributions across the 90° domain-walls in an aberration-corrected STEM at high angle annular dark field (HAADF) mode. We have directly observed not only positively charged but also negatively charged 90° domain-walls at atomic scale.

## Results

PbTiO_3_/SrTiO_3_ multilayer films were prepared by pulsed laser deposition (PLD). The films were deposited on GdScO_3_ (GSO) substrate, which exert tensile strain on the epitaxial PbTiO_3_/SrTiO_3_ superlattices^38^. Such ferroelectric/paraelectric heterostructures have attracted lots of interests because they offer a huge space for exploring the subtle interplay between their multiple order parameters^39^,^40^,^41^.

### Identification of 90° positively-charged-domain-wall

**Figure 1:**
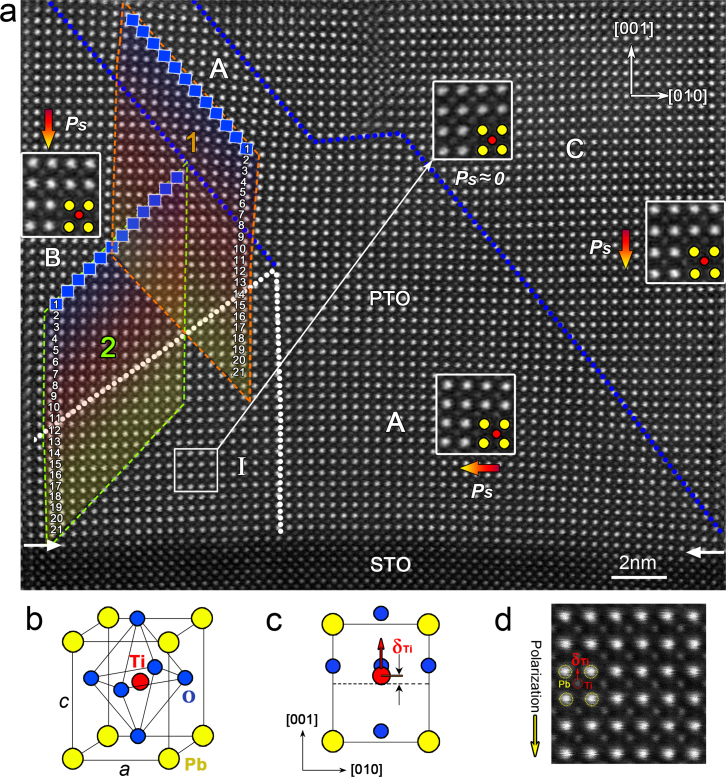
Aberration-corrected HAADF images showing the 90° domain-walls in PbTiO_3_ film and electric dipoles formed by the relative displacements of the Ti^4+^ and Pb^2+^ cation columns. (a) HAADF images where the horizontal arrows denote the interface between the PbTiO_3_ and SrTiO_3_ layers. The blue dotted line traces the ‘head-to-tail' 90° domain-walls, while the white dotted line denotes the area with ‘head-to-head' 90° domain-wall (*area* I). The colored arrows denoted by ‘*Ps*' show the directions of the polarization of PbTiO_3_ beside the 90° charged-domain-wall. The insets show magnifications of the dipoles formed by the displacements of ions in corresponding domains (yellow: Pb^2+^, red: Ti^4+^). Note that, in some PTO units within the *area* I, no obvious displacement of the Ti^4+^ and Pb^2+^ columns are observed, indicating that the polarizations in these unit-cells are restrained. (b) Schematic perspective view of the unit-cell of ferroelectric PbTiO_3_. (c) Projection of the unit-cell along the [100] direction showing the δ*_Ti_*. (d) A representative HAADF image of the dipoles in PbTiO_3_ crystal. The positions of Pb^2+^ columns are brighter than those of the Ti^4+^ columns. δ*_Ti_* is also marked, which is opposite to the spontaneous polarization direction of the PbTiO_3_ denoted by the yellow arrow.

Figure 1a shows an atomic resolution HAADF image of a PbTiO_3_ (PTO) layer near the PbTiO_3_/SrTiO_3_ (PTO/STO) interface. The four insets are magnification of images overlying the respective areas. Yellow cycles denote Pb^2+^ columns and red cycles denote Ti^4+^ columns. O^2−^ columns are invisible due to its weak scattering effects of electrons. As illustrated by the schematic diagrams in figure 1b and c, the atom arrangement of ferroelectric PTO exhibits shifts of the atoms with respect to the cubic perovskite structure. Both the Ti^4+^ and O^2−^ columns are shifted upward (along the [001] direction) towards the upper Pb^2+^ positions and away from the respective lower ones, but the O^2−^ columns are shifted more strongly. As a consequence, a charge dipole forms in the PTO unit-cell because of the separation of negative (O^2−^) and positive (Ti^4+^ and Pb^2+^) charges, as seen by means of coherent high-resolution imaging with negative Cs technique^14^. This dipole defines the direction of the vector of spontaneous polarization *Ps* (pointing from net negative to net positive charge and thus is opposite with the displacement of Ti^4+^, Fig. 1d) parallel to [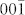]. Thus the atomic resolution HAADF imaging technique could be employed to record the electric dipoles unit-cell by unit-cell (Fig. 1d). The *Ps* direction (yellow arrow in Fig. 1d, colored arrows in Fig. 1a) of PTO unit-cells can be determined from the displacement of Ti^4+^ (δ*_Ti_*) measured in the atomic resolution HAADF image. A careful inspection reveals that in figure 1a there are three polarization domains: lower right, domain A; middle left, domain B; upper right, domain C. The strong tetragonal nature of PTO (*a* = 0.389 nm, *c* = 0.414 nm, ref. 38) makes remarkable difference between scalings of *a* and *c* domains (In Fig. 1a, domain A is an *a* domain, domain B and C are *c* domains), thus domain A is shorter than domain B and C on both sides. This results in a complex strain state near the coherent PTO/STO interface which makes a bending of the STO lattice. The changing of one domain to another gives rise to the switching of polarization vector by 90°. Domain C has the same *Ps* direction as domain B. The position of these 90° domain-walls (DWs), indicated by the blue dotted lines, can be determined directly by mapping the δ*_Ti_* vectors of each PTO unit-cells. It is of interest to notice that the left 90° domain-wall terminates within the PTO matrix, this scenario is suggestive of some unusual dipole behaviors at the bottom left corner of figure 1a, because the *Ps* directions in domain A and B have to encounter each other. Thus, a 90° charged-DW (CDW) is identified, with a ‘head-to-head' arrangement of *Ps* vectors. Such a 90° CDW is actually a broad area (labeled *area* I, as will be specified in the following) and thus is marked by two white dotted lines (Fig. 1a). Nominally, the ‘head-to-head' arrangement of *Ps* produces positive bound charges near the CDW^42^, so here we name it in terms of 90° positively-charged-domain-wall (PCDW). Similarly, the 90° uncharged-domain-wall is short-written as 90° UCDW.

**Figure 2:**
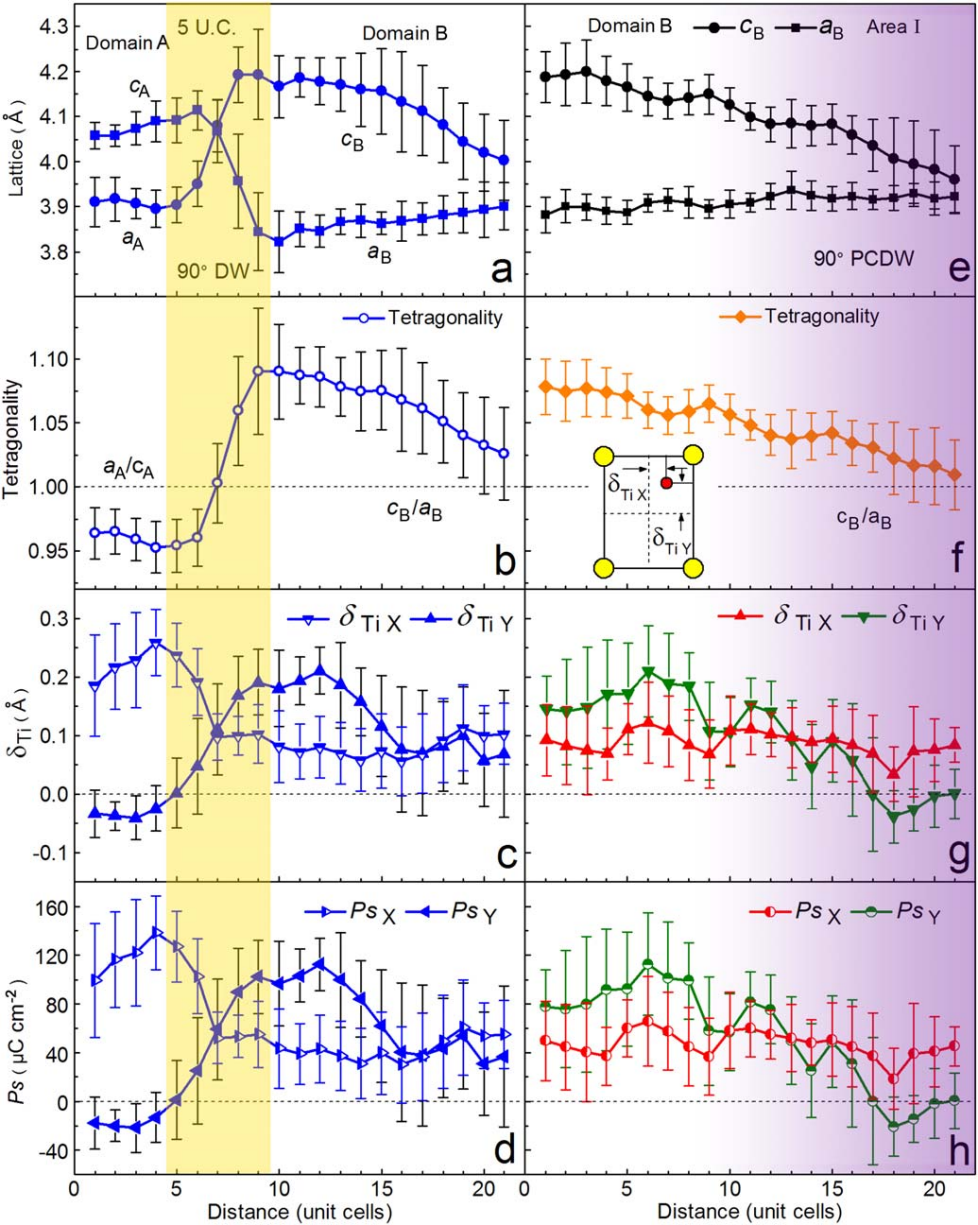
Quantitative analysis of the structural and electric parameters of the 90° PCDW and 90° UCDW. The structural and electric parameters were averaged along {110} (pseudo-cubic) to insure the statistical analysis is parallel to the DWs. (a)–(d) Lattice parameters, tetragonality, δ*_Ti_* and *Ps* extracted from the rhombus-highlighted area marked with ‘1' in Fig. 1a. (e)–(h) Lattice parameters, tetragonality, δ*_Ti_* and *Ps* extracted from the rhombus-highlighted area marked with ‘2' in Fig. 1a. The error bars show the standard deviation with respect to averaging along {110}PTO for each {110} atomic row. The inset in (f) shows the schematic definition of out-of-plane (δ*_TiY_*) and in-plane (δ*_TiX_*) components of the δ*_Ti_*, respectively.

A careful observation indicates that, on both structural and electric level, the 90° PCDW is rather wider than the 90° UCDW. According the famous Kittel's law, the DW width is a crucial factor for determining the DW patterns and thus the properties such as nonlinear electro-optics^3^. The local behaviors of the present 90° PCDW (rhombus-highlighted area labeled ‘2' in the lower left of figure 1a) are comparatively studied with the 90° UCDW (rhombus-highlighted area labeled ‘1' in the upper left of figure 1a), and the average data are obtained along PTO{110}_p_ (subscript p denotes pseudo-cubic), which is generally thought to be the location of 90° DWs in tetragonal ferroelectrics^22^,^23^,^24^. The magnitudes of *Ps* vectors are determined by corresponding δ*_Ti_* because the relationship between the δ*_Ti_* and the *Ps* is well-known^33^. The image drifting are calibrated with reference to the lattice parameter (*a* = 0.5488 nm, ref. 43) of the orthorhombic GSO substrate (not shown here). The structural and polar characters of the 90° UCDW at the upper left of figure 1a (The wall that separates domain A and domain B) are shown in figure 2a–d.

It is seen that the changing of lattice and polarization happens rapidly across the 90° UCDW (Fig. 2a–d), which exhibits the twin-walls character in tetragonal ferroelectrics^9^. The DW thickness is about 5 unit-cells (yellow shadowed in Fig. 2a–d) derived from the measurements of the local structural (lattice and tetragonality in Fig. 2a,b) and electric (δ*_Ti_* and *Ps* in Fig. 2c,d) changes. In comparison, the structural and polar characters across the 90° PCDW are measured, shown in figure 2e–h, which correspond to the lower left area in figure 1a (The wall that separates domain B and *area* I, across the left white dotted line). Amazingly, the changing of both lattice and polarization is much slowly across the 90° PCDW. From domain B to *area* I, the out-of-plane lattice (*c*_B_) continuously changes from 0.42 nm to 0.395 nm without a sharp jump, while the in-plane lattice (*a*_B_) almost kept constant of about 0.39 nm (black solid circles and squares denoted in Fig. 2e). The ratio of *c*_B_/*a*_B_ indicates a slow changing of tetragonality, from 1.075 to 1.01 (Fig. 2f). There is no intersection of *c*_B_ and *a*_B_ lattice, and thus no sudden jump of tetragonality, which displays some unusual features of the 90° PCDW compared with the uncharged domain-walls. Generally, the *Ps* and strain in ferroelectric materials are coupled^8^,^33^, as a result, the *Ps_Y_* also changes slowly from domain B (about 80–100 μCcm^−2^) to *area* I (0) (olive-circle curve in Fig. 2h). However, *Ps_X_* almost keeps almost a constant value of about 40 μCcm^−2^ (red-circle curve in Fig. 2h). It is proposed that the accumulated bound charges induced by the 90° PCDW might be responsible for the invariable *Ps_X_*. The continuous and slow changing of lattice and *Ps* indicate that there is no obvious DW across the domain B and *area* I (violet gradual shadows in Fig. 2e–h). In other words, here the 90° PCDW is a broad area where the *Ps* vectors are disordered.

**Figure 3:**
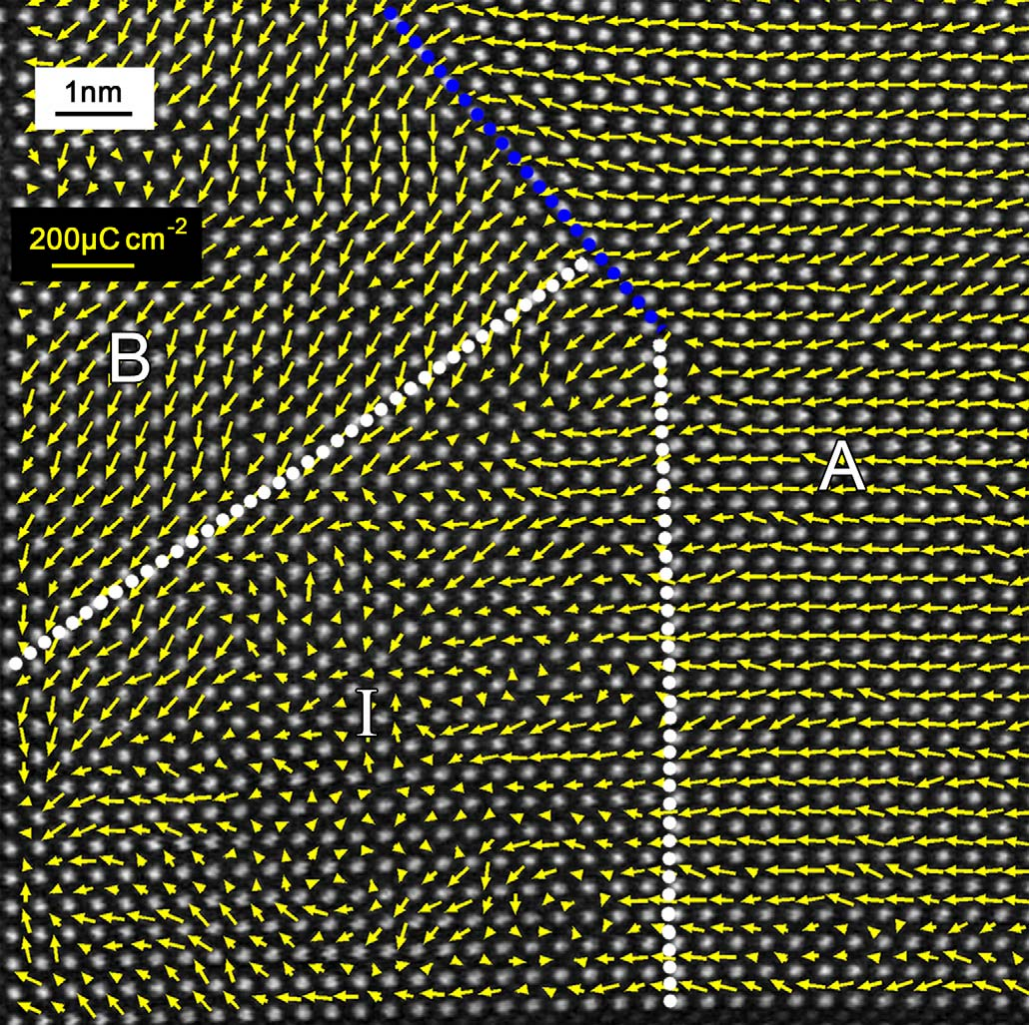
*Ps* vector mapping across the 90° PCDW and 90° UCDW in Fig. 1a. *Ps* vectors calculated though the δ*_Ti_* of both the 90° PCDW and 90° UCDW away from PTO/STO interface was shown. The blue dotted line denotes the 90° UCDW, the white dotted lines embrace the 90° PCDW area (*area* I in Fig. 1a). Note the restriction and disorder of the *Ps* vectors at the 90° PCDW, signifying that there is no obvious ‘domain-wall' at the encounter of the *Ps* vectors. The length of the yellow arrows represents the modulus of the *Ps* with respect to the yellow scale bar in the upper left corner.

To directly gain insight into the polarization distributions, the *Ps* vectors of each unit-cell near the 90° PCDW were mapped and superimposed on the HAADF image (Fig. 3). The arrows located at the Ti^4+^ column positions indicate the directions and modulus of the *Ps* vectors. For most PTO cells, the *Ps* values are in the range of 70–100 μCcm^−2^. Unlike the 90° UCDW, where the directions of the *Ps* vectors changed rapidly, the *Ps* is strongly restricted and disordered at the 90°PCDW. According to the disorder of the *Ps* vectors, no obvious ‘domain-wall' could be identified, which is consistent with its lattice behavior (Fig. 2e, f). Bound charges produced by the ‘head-to-head' dipole arrangement may be responsible for the restriction and disorder of the 90°PCDW.

### Identification of 90° negatively-charged-domain-wall

In addition to the 90° PCDW, 90° *Ps* vectors configured as ‘tail-to-tail' CDW is also identified in the present films. Since the ‘tail-to-tail' *Ps* arrangement may nominally induce negative bound charges near the CDW^42^, here we name it as 90° negatively-charged-domain-walls (90° NCDW). We will see that the structural and electric parameters of 90° NCDW are remarkably different from those of 90° PCDW.

**Figure 4:**
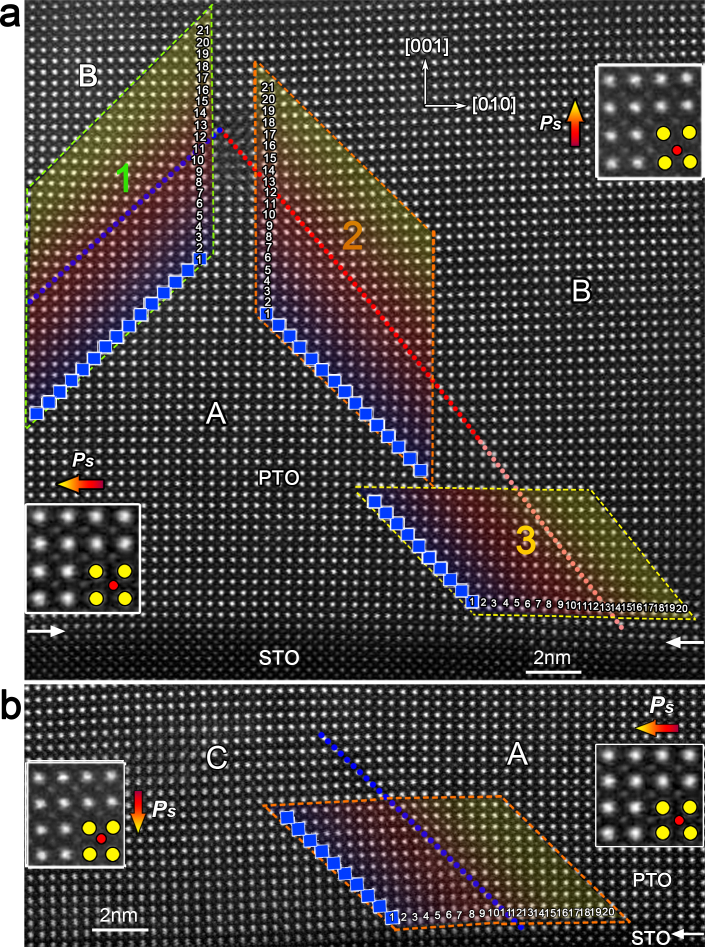
Aberration-corrected HAADF imaging of the electric dipoles formed near the 90° NCDW in PbTiO_3_. (a) A PbTiO_3_ film near a SrTiO_3_ layer shows both charged and uncharged 90° domain-walls. The blue dotted line traces the 90° UCDW, the red dotted line traces the upper 90° NCDW, while the light red dotted line traces the lower 90° NCDW near the STO/PTO interface. The insets are magnifications of the dipoles formed by the displacements of ions in corresponding domains. (b) A PbTiO_3_ film near a SrTiO_3_ layer shows 90° UCDW (blue dotted line) near the PTO/STO interface. The insets are magnifications of the dipoles formed by the displacements of ions in corresponding domains. This 90° UCDW is the connection between domain A (in fig. 4a) and a *c* domain (named as ‘domain C', as illustrated). Domain C was located at the left side of domain A (fig. 4a). The atomic images in rhombus-highlighted area is used to extract lattice parameters, tetragonality, δ*_Ti_* and *Ps*.

**Figure 5:**
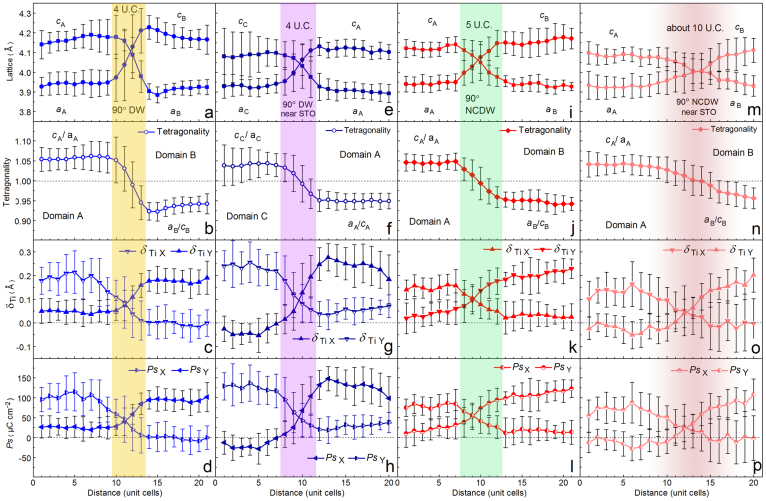
Quantitative analysis of the structural and electric parameters of the 90° NCDW and 90° UCDWs. (a)–(d) Lattice parameters, tetragonality, δ*_Ti_* and *Ps* extracted from the rhombus-highlighted area marked with ‘1' in Fig. 4a. (e)–(h) Lattice parameters, tetragonality, δ*_Ti_* and *Ps* extracted from a 90° UCDW near the PTO/STO interface (the rhombus-highlighted area in Fig. 4b). (i)–(l) Lattice parameters, tetragonality, δ*_Ti_* and *Ps* extracted from the rhombus-highlighted area marked with ‘2' in Fig. 4a. (m)–(p) Lattice parameters, tetragonality, δ*_Ti_* and *Ps* extracted from the rhombus-highlighted area marked with ‘3' in Fig. 4a. The error bars show the standard deviation with respect to averaging along {110}PTO for each {110} atomic row. Note that the 90° UCDWs near the PTO/STO interface and away from the interface share the same characteristics of a sharp jump of structural and electric behaviors. In contrast, the structural and electric parameters of the lower 90° NCDW near the interface are remarkably different from those away from the interface.

Figure 4a shows an atomic resolution HAADF image of a PTO layer containing twin structures. Using the same methodology as that in figure 1, the position of 90° UCDWs is outlined, as marked by the blue dotted lines in the upper left part of figure 4a. By mapping the δ*_Ti_* vectors of each PTO unit-cell, 90° DW with ‘tail-to-tail' arrangement of *Ps* vectors is identified, as traced by the red (upper segment) and light red (lower segment near the PTO/STO interface) dotted lines. The 90° NCDW separates domain A and B in figure 4a, with *Ps* directions pointing to left and top, respectively (magnified insets in Fig. 4a). Different from the 90° PCDW, the upper segment of the 90° NCDW is much narrower like that of the uncharged ones, while the lower segment is broadened similar to that of the 90° PCDW. The local behaviors of the two segments of the 90° NCDW are comparatively analyzed with the 90° UCDWs. In figure 5, the upper 90° NCDW (rhombus-highlighted area labeled ‘2' in Fig. 4a) is compared with the left 90° UCDW (rhombus-highlighted area labeled ‘1' in Fig. 4a), while the lower 90° NCDW (rhombus-highlighted area labeled ‘3' in Fig. 4a) is compared with a 90° UCDW which is also near the PTO/STO interface neighboring with the 90° NCDW, seen in figure 4b. Here this 90° UCDW is the connection between domain A and a *c* domain, which was located at the left side of domain A. The lattice and *Ps* characters of the left 90° UCDW (Fig. 4a) as well as the 90° UCDW near the PTO/STO interface (Fig. 4b) are shown in figure 5a–d and figure 5e–h. We can see that the width of 90° UCDW was not disturbed by the PTO/STO interface (Fig. 5e–h), since both the structural and electric parameters changed rapidly across these 90° UCDWs. However, the lattice parameters of these two 90° UCDW are different: the *c*-axis in domain A and B in figure 5a (0.415–0.42 nm) are bigger than those in domain C and A in figure 5e (about 0.41 nm). These differences were probably induced by the complex strains near the PTO/STO interface^33^. According to the sharp change of lattice and *Ps* vectors, both the 90° UCDWs possess the same width about 4 unit-cells, which is qualitatively the same as the 90° UCDW in figure 2a.

The structural and electric parameters of the upper and lower segments of the 90° NCDW were analyzed (Fig. 5i–l and Fig. 5m–p). The lattice and polarization also change rapidly across the upper 90° NCDW. This DW width is about 5 unit-cells, almost the same as the 90° UCDW, shadowed in light blue in figure 5i–l. However, for the lower segment of the 90° NCDW, the corresponding parameter slopes are obviously abated. The width of this DW is about 10 unit-cells (gradually shadowed in red in Fig. 5m–p), which is much bigger than that of the 90° UCDW. Nevertheless, like the 90° UCDW, the location of the 90° NCDW is still along {110} PTO.

**Figure 6:**
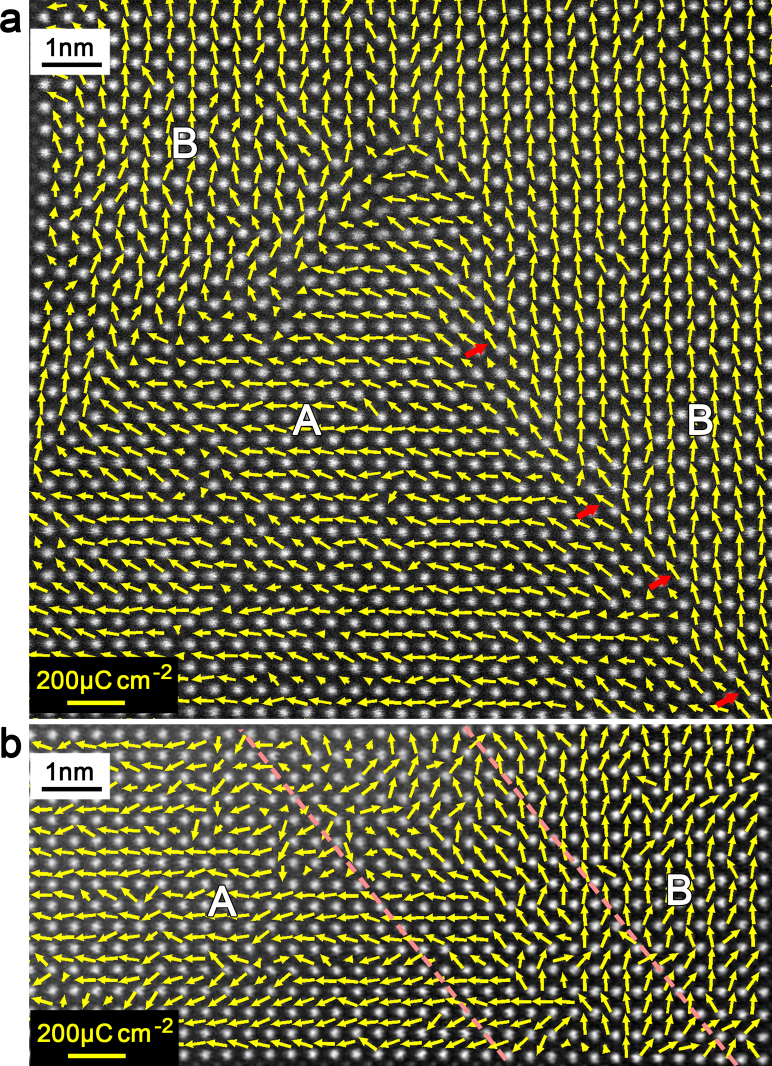
Mappings of the *Ps* vectors across the 90° NCDW and 90° UCDW. (a) *Ps* vectors of 90° UCDW and 90° NCDW away from PTO/STO interface. Note the bifurcation of the *Ps* and the trend of diagonal *Ps* directions (marked with red arrows) across the 90° NCDW. (b) *Ps* vectors across the 90° NCDW near the PTO/STO interface. It is seen that the scattering of the *Ps* vectors at this 90° NCDW makes the “wall” into a “band”. In (a) and (b), the length of the yellow arrows represents the modulus of the *Ps* with respect to the yellow scale bar in the lower left corner.

The *Ps* vectors of each unit-cell near the 90° NCDW are also mapped and superimposed on the HAADF image (Fig. 6a, b). The 90° UCDW and the upper segment of the 90° NCDW are shown in figure 6a. Unlike the 90° UCDW, where the *Ps* directions rotate sharply with 90° at the DW, the *Ps* is almost maintained and rotates inchmeal at the 90° NCDW. The *Ps* vectors, in one or two units away from the 90° NCDW, are somewhat inclined to the NCDW both in domain A and B. And this trend is most obvious at the 90° NCDW because the *Ps* vectors rotate about 45° respective to horizontal or vertical plane (marked with red arrows in Fig. 6a), which produces diagonal *Ps* directions along the 90° NCDW. This inclination of *Ps* vectors along the 90° NCDW may help to relieve the bound charges and thus lower the depolarization field^3^, and to stabilize the narrow 90° NCDW. In contrast, the *Ps* vectors seem to be strongly disturbed at the lower segment of the 90° NCDW near the PTO/STO interface, as shown in figure 6b. According to the diffusion of the *Ps* vectors, no obvious ‘domain-wall' could be identified. Instead, it is more like a ‘domain-wall-band' with a thickness about 10 unit-cells, which is consistent with the lattice behaviors (Fig. 5m, n). Except for the bound charges, the PTO/STO interface may also be responsible for the diffusion of the lower segment of the 90° NCDW because of the interface-induced depolarization field which is very common in thin film ferroelectrics^3^.

**Figure 7:**
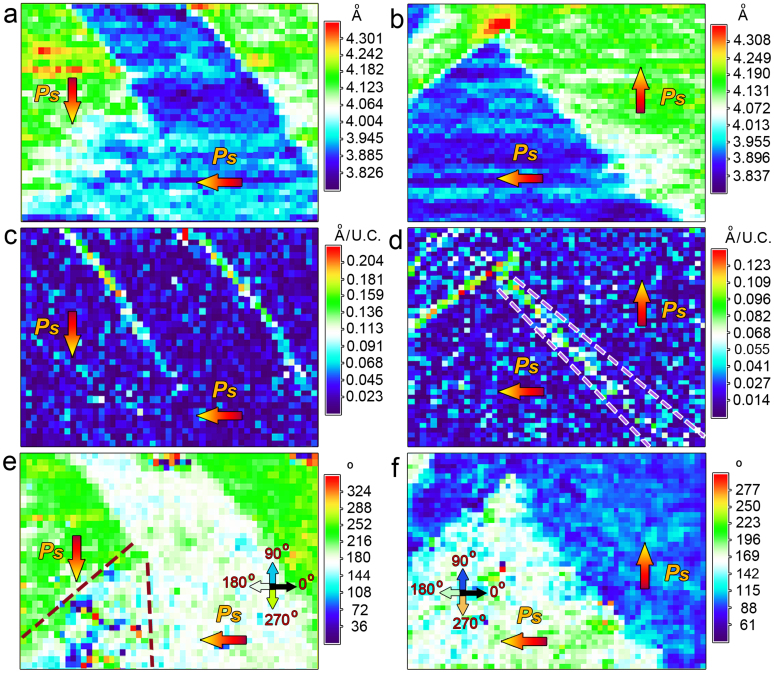
2-D mappings of structural and electric behaviors showing the differences between 90° CDWs and 90° UCDWs. (a, b) Out-of-plane lattice spacing mapping for the 90° PCDW and NCDW. (c, d) Lattice gradient mappings (lattice gradient of the out-of-plane lattice mappings along in-plane direction, mapped unit-cell by unit-cell) for the two types of domains. Note the alleviated lattice gradient across the 90° CDWs, especially of the 90° PCDW, compared with sharp lattice gradient across the 90° UCDWs. (e, f) *Ps* angle mappings for the two types of domains. The definition of 0°, 90°, 180°, and 270° are marked with colored arrows with corresponding color-scale. In case of the 90° PCDW, the *Ps* angles are markedly disordered.

To directly visualize the 2D structural parameters and *Ps* angles, unit-cell-wise structure and *Ps* angle mapping are displayed. The lattice parameter, gradient of the lattice parameter and *Ps* angles of the PTO unit-cells near the 90° PCDW and NCDW are mapped unit-cell by unit-cell, as shown in Figure 7. The out-of-plane lattice spacing mapping results clearly exhibit the 90° UCDWs, since there is sudden jump of the lattice spacing (blue to green, presumably corresponds to 0.39–0.42 nm, Fig. 7a, b). Although the 90° PCDW is diffused since the lattice spacing slowly changes from 0.395 to 0.42 nm (light blue to white then to green) with a width about several tens unit-cells (Fig. 7a, which is consistent with Fig. 2e), we note that the 90° NCDW is much sharper (Fig. 7b). To visually show the differences among the uncharged, positively and negatively-charged 90° DWs, in-plane lattice gradient of the out-of-plane lattice spacing are mapped. The lattice gradient is defined as |*c*_x+1_ − *c*_x_|/1U.C., where *c*_x_ denotes an out-of-plane lattice spacing and *c*_x+1_ denotes the out-of-plane lattice spacing of the right neighbor unit-cell of *c*_x_, and ‘unit-cell' is abbreviated as ‘U.C.', (Fig. 7c, d). The uniform dark blue means there is no in-plane lattice gradient since there is no lattice change in a single domain. The sudden change of lattice *c* to *a* (or *a* to *c*) across the 90° UCDWs makes obvious contrast in figure 7c, d. The maximum of the lattice gradient is about 0.01 to 0.015 nm/U.C. across the 90° UCDWs. It is clear that the left 90° UCDW in figure 7c terminates in the matrix, which results in the formation of the 90° PCDW. Compared with the uncharged DWs, the lattice gradient of the 90° PCDW is invisible (Fig. 7c), this means the lattice change across the 90° PCDW is much slower. Such a status is also seen in figure 2e. However, the 90° NCDW possesses visible lattice gradient (Fig. 7d). Moreover, the lattice gradient of the upper segment is comparable to the 90° UCDW (note that their color-scales are almost the same). Nevertheless, as seen in figure 7d, the color-scale of the 90° NCDW changes gradually from green to light blue as the DW tracing from top to bottom. This indicates that when the 90° NCDW reaches the PTO/STO interface, the lattice gradient is continuously relieved. In addition, the DW is broadened simultaneously with relief of the lattice gradient, as marked with the violet dotted lines (Fig. 7d). Such a status is also seen in figure 5m.

The *Ps* angles of each unit-cell near the 90° PCDW and 90° NCDW are also mapped (Fig. 7e, f). It is seen that the *Ps* directions change rapidly across the 90° UCDWs, behaving like their lattice gradient. However, the *Ps* directions at the 90° PCDW are strongly disordered (see the color fluctuation in Fig 7e). Moreover, this wedgy disordered area is much broader than the uncharged ones with tens of unit-cells at the bottom. For the 90° NCDW, the change of *Ps* angles are much sharper than those of the 90° PCDW. This is almost comparable to the 90° UCDWs for the upper segment and just somewhat relieved for the lower segment (Fig. 7f).

## Discussion

Although the 180° CDWs are common in uniaxial^42^,^44^ and possible in multiaxial ferroelectrics^14^,^45^, the 90° CDW in tetragonal ferroelectrics in our experiments is the first direct atomic-scale observation. Generally, it is proposed that internal charge carriers, for example, oxygen vacancies or electron holes, can screen the bound charges accompanying with the CDW^3^,^42^,^45^,^46^, thus the CDW could be stabilized. In the case of present PTO/STO films, it is proposed that the charge carriers are probably oxygen vacancies (Vo^2+^), which possess positive charges screening the negative bound charges induced by the 90° NCDW. This inference is constant with our observation because the 90° NCDW is less disturbed compared with the 90° PCDW, and the later probably deserved a depletion layer of Vo^2+^, which was repulsed by the positive bound charges^44^. In addition, for ferroelectrics with a specific charge carriers (positive, such as Vo^2+^ and electron holes, or negative, such as electrons), only one kind of CDW can be effectively screened^42^,^44^. On this condition, if a ferroelectric possesses both PCDW and NCDW simultaneously, only one kind of CDW could be neutralized, that is probably the situations of our present observation, which was proposed in theoretical work^42^. Our results suggest the lattice and polarization are coupled ideally for all kinds of 90° DWs in PTO. This is remarkably different from the decoupling observed by AFM and PFM in BaTiO_3_ (Refs. 47) which showed the polarization distribution is much wider than that of lattice.

The charge carrier accumulation was hypothesized to be a cause for the increased conductivity at the CDWs in ferroelectrics^3^,^44^. In hexagonal ferroelectric HoMnO_3_, the widths of 180° PCDW and NCDW were also found to be different, based on the conductive atomic force microscopy (cAFM) observation where the DW width was absent from atomic-scale information limited by the AFM tip radius. In tetragonal PbTiO_3_ films of the present study, the difference between the 90° PCDW (carrier depletion) and NCDW (carrier accumulation) at atomic scale may qualitatively explain the different conduction behaviors between ‘head-to-head' and ‘tail-to-tail' 180° DWs in HoMnO_3_ (ref. 44). In addition, the coupling of *Ps* and elastic strain for the 90° DWs is much stronger than the 180° DWs^8^,^10^,^11^,^12^. This implies that, during the 180° switching the 90° CDWs may generally exist, at least in a dynamical style. Earlier PFM studies showed that this kind of 90° CDWs may play an important role for the retention failure of the PZT memories because the switched 180° domains could be reversed by the 90° CDW as time elapses^48^,^49^.

In summary, by using aberration-corrected STEM, the unusual frustration of dipole arrangements and strain behaviors of 90° CDWs in PTO/STO multilayer films are identified on the atomic-scale, where the widths, polarization distributions, and strains across these charged domain walls are mapped quantitatively. “Glass-like” dipole behaviors are observed at the 90° CDWs. We anticipate the present atomic-scale investigations of the uncharged and charged 90° DWs may help to interpret the switching behaviors, the newly realized domain-wall functions, and the retention failure mechanism in ferroelectrics. Moreover, the present study is expected to clarify the long-standing argument about the width of the 90° DWs in tetragonal ferroelectrics. During the review stage of this paper, a research group in Michigan University reported an occurrence of 90° charged domain walls in Pb(Zr_0.2_Ti_0.8_)O_3_ thin films formed by in-situ electric response^50^.

## Methods

### Thin-film synthesis and STEM sample preparations

The PbTiO_3_/SrTiO_3_ thin films were deposited on GdScO_3_ substrates by pulsed laser deposition (PLD), using a Lambda Physik LPX 305i KrF (λ = 248 nm) excimer laser. The PbTiO_3_ targets were 3 mol% Pb-enriched sintered ceramics. The target-substrate distance was 40 mm. The background pressure was 10^−5^ Pa. During the growth of PbTiO_3_, the substrate temperature was kept at 650°C, with a laser energy density of 2 Jcm^−2^, a laser repetition rate of 5 Hz and under an oxygen pressure of 20 Pa. For the growth of SrTiO_3_ layers, the substrate temperature was also 650°C, with a laser energy density of 1 Jcm^−2^, a laser repetition rate of 2 Hz and under an oxygen pressure of 8 Pa. Before deposition, the GdScO_3_ substrate was pre-heated at 750°C for 5 min to clean the substrate surface and then cooled down to the growth temperature (10°C/min). The laser was focused on the ceramic target for 30 min pre-sputtering to clean the target surface. After deposition, the film was in-situ-annealed at 650°C in an oxygen pressure of 5 × 10^4^ Pa for 10 min, and then cooled down to room temperature at a cooling rate of about 5°C/min. The samples for the STEM experiments were prepared by slicing, gluing, grinding, dimpling, and finally ion milling. A Gatan PIPS was used for the final ion milling.

### STEM imaging and analysis

One of the great advantages of HAADF-HRSTEM imaging mode is that it is not sensitive to the variety of local specimen thickness, and therefore, it is quite suitable for large-scale imaging. The finding of the novel domain configurations in the relatively large scale in this work is believed to benefit from the HAADF imaging mode. In addition, the aberration-corrected TEM used in this study features very little drift; for example, the STEM spot drift and specimen drift are 0.14 nm/min and 0.16 nm/min, respectively. HAADF images in this study were recorded using aberration-corrected scanning transmission electron microscopes (Titan Cubed 60–300 kV microscope (FEI) fitted with a high-brightness field-emission gun (X-FEG) and double Cs correctors from CEOS, and a monochromator operating at 300 kV). The convergence angle of the electron beam is 25 mrad, yields a probe size of less than 0.10 nm. The determination of the atom coordinates in the HAADF-STEM images were carried out by fast Fourier transform (FFT) filtering the images using only a low-pass annular mask restricted slightly more than the resolution limit of the image, thus the lattice spacing and Ti^4+^ shifts (δ*_Ti_*) were deduced. The atom positions were determined accurately by fitting them as 2D Gaussian peaks by using Matlab^14^,^33^,^34^,^51^. The δ*_Ti_* were calculated as a vector between each Ti^4+^ and the center of mass of its four nearest A-site neighbor Pb^2+^. The *Ps* vectors were deduced by the δ*_Ti_*. The visualization of the 2D *Ps* vectors (Fig. 3 and Fig. 6) was carried out using Matlab. The visualization of the lattice, lattice gradient and *Ps* angles (Fig. 7) was carried out using the combination of Matlab and ImageJ software^34^.

## Author Contributions

The project of interfacial STEM characterization in oxides was conceived by Y.L.Z. and X.L.M.; thin film growth, TEM specimen preparation and STEM observations were performed by Y.L.T.; Y.J.W. participated digital analysis of the HAADF-STEM images; W.Y.W., Y.B.X., W.J.R. and Z.D.Z. have contributions in thin film growth; Y.L.T., Y.L.Z. and X.L.M. jointly interpreted the data and wrote the paper.
